# Activation of STAT transcription factors by the Rho-family GTPases

**DOI:** 10.1042/BST20200468

**Published:** 2020-09-11

**Authors:** Jessica Corry, Helen R. Mott, Darerca Owen

**Affiliations:** Department of Biochemistry, University of Cambridge, 80 Tennis Court Road, Cambridge CB2 1GA, U.K.

**Keywords:** Cdc42, G-proteins, Rac, Rho-family, signal transducers and activators of transcription, transcription factors

## Abstract

The Rho-family of small GTPases are biological molecular switches that are best known for their regulation of the actin cytoskeleton. Through their activation and stimulation of downstream effectors, the Rho-family control pathways involved in cellular morphology, which are commonly activated in cancer cell invasion and metastasis. While this makes them excellent potential therapeutic targets, a deeper understanding of the downstream signalling pathways they influence will be required for successful drug targeting. Signal transducers and activators of transcription (STATs) are a family of transcription factors that are hyper-activated in most cancer types and while STATs are widely understood to be activated by the JAK family of kinases, many additional activators have been discovered. A growing number of examples of Rho-family driven STAT activation, largely of the oncogenic family members, STAT3 and STAT5, are being identified. Cdc42, Rac1, RhoA, RhoC and RhoH have all been implicated in STAT activation, contributing to Rho GTPase-driven changes in cellular morphology that lead to cell proliferation, invasion and metastasis. This highlights the importance and therapeutic potential of the Rho-family as regulators of non-canonical activation of STAT signalling.

## Introduction

**Figure 1.**
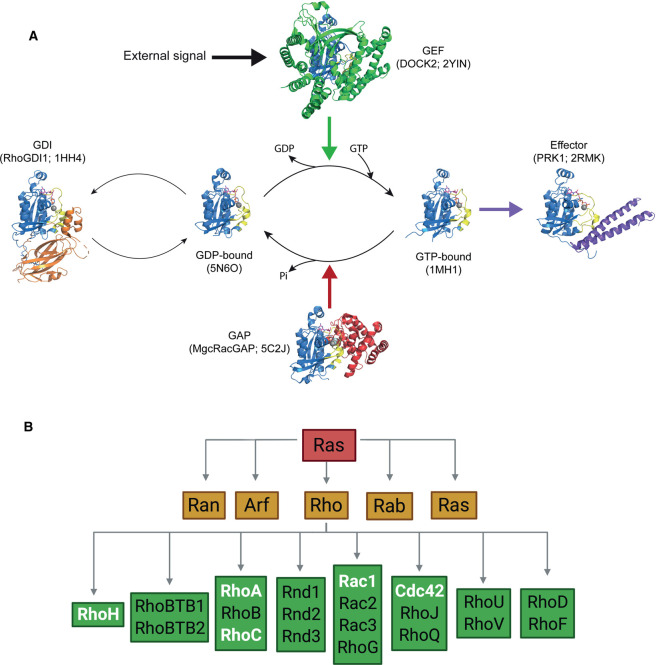
Introduction to the Rho-family of GTPases. (**A**) The GTPase cycle of Rho-family small G proteins. Rac1 is activated when an external signal leads to GEF proteins such as DOCK2 binding, leading to stabilisation of the nucleotide-free state. The subsequent binding of GTP allows effectors to bind to the Rac1 protein. Binding of GAP proteins such as MgcRacGAP, stimulate the intrinsic GTPase activity to regenerate the GDP-bound form. RhoGDI binds to Rac1·GDP and, by sequestering the C-terminal geranylgeranyl group, removes the G protein from membranes. In each cartoon, the GTPase is shown in blue, with the switch 1 and switch 2 regions coloured yellow, the nucleotide shown as sticks and the Mg^2+^ ion as a grey sphere. The PDB codes of all structures are shown; the MgcRacGAP is in complex with Cdc42, all other structures are of Rac1. (**B**) The Ras superfamily of small GTPases separates into five families: Ran, Arf, Rho, Rab and Ras. The Rho-family further divides into several subfamilies. Family members that are the subject of discussion in this review are highlighted in white.

The Ras superfamily of small GTPases comprises over 150 biological molecular switches. These proteins are capable of switching between an inactive, GDP-bound state and an active, GTP-bound conformation that transduces signals via downstream effector proteins. The GTPase cycle is influenced by a variety of regulators (Figure 1A). GTPase activating proteins (GAPs) stimulate intrinsic GTPase activity, thereby promoting the hydrolysis of GTP to GDP and deactivating the switch. Guanine nucleotide exchange factors (GEFs) instead promote the release of GDP, allowing for the binding of free GTP to promote switch activation. Additionally, guanine nucleotide-dissociation inhibitors (GDIs) promote the inactive conformation by inhibiting the release of GDP and sequestering the GTPase away from membranes [1].

The Ras superfamily separates into five branches that influence distinct cellular processes (Figure 1B). The Ras-family control cell survival and proliferation, the Rab- and Arf-families regulate vesicle trafficking, Ran coordinates nucleocytoplasmic transport and the Rho-family regulate the actin cytoskeleton [2]. The prototypical members of the Rho-family include: Cdc42, which controls filopodia formation and actin stabilisation; Rac1, which regulates lamellipodia formation and membrane ruffling and RhoA, which controls actin stress fibre formation [2]. Once activated, small GTPases relay signals downstream via effector proteins, of which at least 70 are known for the Rho-family, allowing Rho-protein activation to modulate many biological pathways [3].

**Figure 2.**
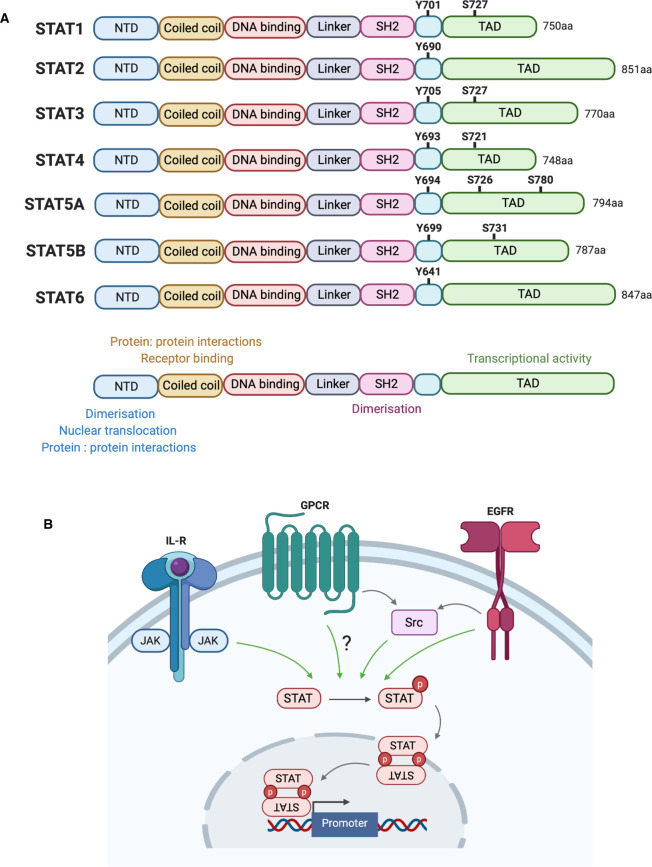
Introduction to the STAT family of transcription factors. (**A**) Homologous STAT family domain structure. All family members contain an N terminal domain (NTD) that is involved in dimerisation, nuclear translocation and interaction with other proteins; a coiled coil domain involved in protein: protein interactions and receptor binding; a DNA binding domain (DBD) that directly binds to target DNA sequences; a linker region; an SH2 domain involved in dimerisation by interacting with pTyr of another activated monomer and a C-terminal transactivation domain (TAD) that regulates STAT transcriptional activity. Total amino acid length of each STAT member is listed. A conserved tyrosine residue is shared between all family members, corresponding to Tyr701 in STAT1. Additionally, some family members share a conserved serine residue in the TAD domain, corresponding to Ser727 in STAT1. (**B**) STAT activation mechanisms. STAT members can be tyrosine phosphorylated by cytoplasmic JAK kinases activated at cytokine receptors, in addition to GPCR activated non-receptor tyrosine kinases like Src and growth factor receptors such as EGFR. Phosphorylation (represented by red circles) allows for reciprocal interactions between pTyr and SH2 domains of monomers that allow dimer formation. Translocation of active dimers to the nucleus allows for binding upstream of target gene promoters.

The signal transducers and activators of transcription (STAT) comprise a seven-member family of transcription factors involved in implementing cytokine and growth factor-induced gene expression [4]. The family respond to a variety of extracellular stimuli and are generally ubiquitously expressed, except for STAT4, which is restricted to the testis, thymus and spleen [5]. The STAT family have a conserved domain architecture (Figure 2A) with a critical conserved tyrosine residue, corresponding to Tyr701 in STAT1, that is required for STAT activation and function [6]. Phosphorylation of this conserved tyrosine residue drives reciprocal interactions between the pTyr of one STAT monomer and the SH2 domain of another, mediating dimerisation (Figure 2B) [7]. The interaction can drive the formation of STAT homodimers or can occur between different STAT family members to produce heterodimers. Additionally, some STAT members have one or more conserved serine residues in the TAD, which are also targeted for phosphorylation by various members of the mitogen-activated protein kinase (MAPK) family [8]. Although the biological significance of these residues is less well understood, phosphorylation at these sites potentially contributes to full STAT activation [8]. Following phosphorylation and dimerisation, STAT dimers translocate to the nucleus and bind to target gene promoters to stimulate transcription (Figure 2B).

Due to the increased activation of their stimulators, STATs are commonly constitutively active in disease and elevated activation of STAT3, STAT5A, STAT5B and in some cases STAT1, has been observed in most cancer types [9]. Canonically, STAT proteins are activated by the JAK family of kinases comprising JAK1/2/3 and TYK2 [4]. However, additional non-canonical STAT activators include: Src, EGFR and kinases/pathways regulated by the Rho-family of GTPases [10,11]. The non-canonical routes of STAT activation by Rho-family GTPases are the subject of this review.

## Cdc42

Cdc42 was first shown to influence STAT family activation when expression of constitutively active Cdc42 mutants in Rat-1, HEK239T and MEF cells increased expression from a STAT3-driven luciferase reporter [12–14]. Expression of active Cdc42 in MEFs also stimulated STAT3 phosphorylation on Tyr705 and Ser727 and nuclear translocation [14]. Similarly, expression of constitutively active Cdc42 has been shown to drive expression of a STAT5A-driven luciferase reporter but in a more restricted, cell line dependent fashion [15].

**Figure 3.**
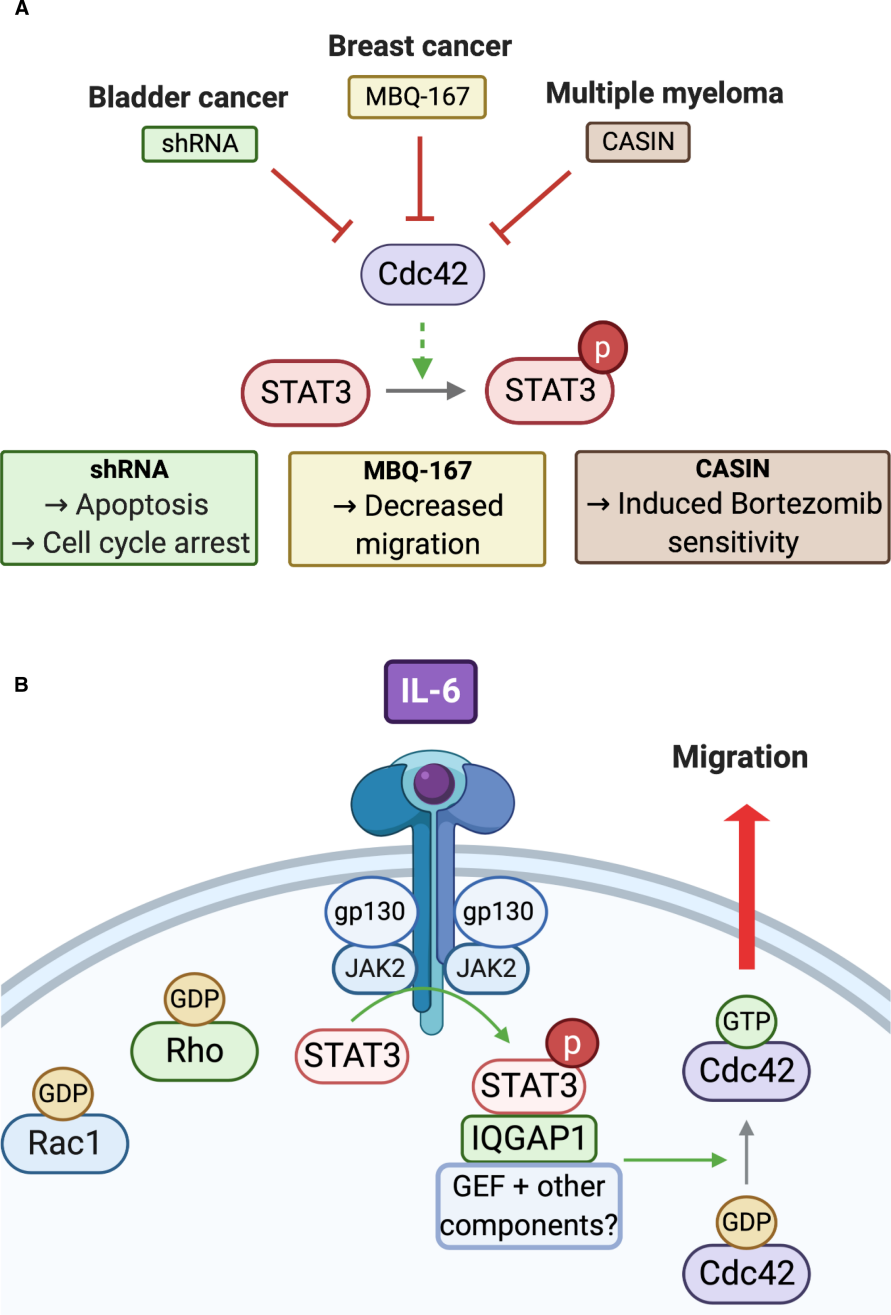
Cdc42-STAT3 in cancer. (**A**) Inhibition of the Cdc42–STAT3 relationship in cancer. Cdc42 mediated activation of STAT3 is described in various cancer types. Drugs that target Cdc42 including CASIN and MBQ-167 along with shRNA mediated knockdown of Cdc42, have all been shown to induce phenotypic changes in cancer cell lines including the induction of apoptosis, reduced migration and drug sensitivity. (B) Cdc42–STAT3 in pancreatic cell migration. In PANC1 pancreatic cancer cells, IL-6 stimulates JAK2-STAT3 activation via the IL6-R and gp130 co-receptor. STAT3 then relays this activation to Cdc42, an effect exclusive to this Rho-family protein. STAT3-driven activation of Cdc42 involves its interaction with IQGAP1, a scaffold protein, and an additional yet unidentified Cdc42 regulator. Cdc42 is stimulated and catalyses the cellular changes that aid PANC1 cell migration.

The cellular consequences of inhibiting the Cdc42–STAT3 signalling axis in cancers have also emerged (Figure 3A). In bladder cancer cell lines, shRNA-mediated knockdown of Cdc42 blocked cell cycle progression and induced apoptosis, which was attributed to the observed reduction in pTyr705 STAT3 levels [16]. In breast cancer, a novel small molecule Rac1/Cdc42 dual inhibitor, MBQ-167, impaired STAT3 activation, resulting in decreased metastatic breast cancer cell migration [17]. Additionally, a role for the Cdc42–STAT3 signalling node has been identified in drug resistant multiple myeloma (MM) cells that are sensitive to loss of Cdc42. Treatment with CASIN, a Cdc42 selective small molecule inhibitor, induced cell death and inhibited drug resistant MM cell proliferation. Bortezomib is a first-in-class proteasome inhibitor and comprises one of the elements of the current standard of care triplet regime for MM. Bortezomib-resistant MM cells have been shown to have elevated Cdc42 expression, in addition to high levels of EGFR and active STAT3. CASIN treatment is thought to sensitise bortezomib resistant cells by blocking Cdc42-dependent STAT3 activation [18].

Additionally, the role of Cdc42–STAT3 in the migration of pancreatic cells was recently presented (Figure 3B). IL-6 was shown to stimulate PANC-1 cell migration and induce morphological changes such as the formation of filopodia, a Cdc42-dependent feature. An increase in Cdc42 activation was revealed in response to IL-6 stimulation, an effect exclusive to this Rho-family protein [19]. IL-6 treated PANC1 cells were also shown to have elevated pTyr705 STAT3 levels, which interestingly was unaffected by Cdc42 inhibition, suggesting that Cdc42 may be acting downstream of STAT3 in this context (Figure 3B). Co-immunoprecipitation and mass spectrometry identified an interaction between STAT3 and IQGAP1, a known Cdc42 binding scaffold protein [19]. siRNA knockdown of IQGAP1 was also shown to impair Cdc42 activation and block the migratory phenotype otherwise observed. The mechanism for IQGAP1's stimulation of Cdc42 is not yet known but must include other known IQGAP1 interactors, presumably including a GEF to promote Cdc42 activation. The authors noted that IL-6 stimulated activation of Cdc42 was not however a universal effect in pancreatic cell lines. DanG cells, a genetically heterogenous pancreatic cell line with differing oncogenic driver mutations to PANC1, also showed an IL-6 dependent stimulation of STAT3 but this was not coupled with Cdc42 activation or cell migration. Taken together, these results suggest that tumour heterogeneity is likely to influence the potential for therapeutic intervention in this newly identified pathway [19].

Finally, one known Cdc42 GEF, DOCK8, has been shown to co-immunoprecipitate with STAT3 in human primary T cells [21]. Additionally, autosomal recessive hyper IgE syndrome (AR-HIES) patients have *DOCK8* mutations that block STAT3 signalling and impair T helper 17 cell differentiation [20,21]. T cells expressing the mutant N1986Y DOCK8, with abolished GEF activity, can no longer amplify cytokine induced STAT3 activation, nuclear import or STAT3 driven transcription [21].

## Rac1

### Rac1 and STAT3/5

Of all Rho-family GTPases, most is currently known regarding the relationship between Rac1 and the STAT family. As with Cdc42, expression of constitutively active Rac1 mutants has been shown to increase expression from both STAT3 and STAT5A-driven luciferase reporters in a variety of cell lines including: Rat1, HeLa, HEK239T, CHO and COS-1 [12,13,15,22]. Conversely, expression of a dominant negative T17N Rac1 mutant in COS-1 cells abolished STAT3 transcriptional activity, as did introduction of a Y705F STAT3 mutant in Rat1 cells in the context of constitutively active Rac1 [12,22]. Initial luciferase reporter studies from multiple groups demonstrated many examples of Rac-dependent induction of STAT transcriptional activity, although this was not a universal effect, with varying degrees of activation observed in different cell lines with different reporter constructs [14]. Multiple studies have also identified induction of Tyr705 and Ser727 STAT3 phosphorylation upon G12V (activated) Rac1 expression, which results in nuclear translocation of STAT3 and DNA binding [12,14,22]. It has been contested whether autocrine activation via the IL-6R is required for Rac1–STAT3 activation, since the addition of neutralising IL-6R antibodies blocked STAT3 nuclear import in one study using HeLa cells but had no effect in an alternative study employing MEFs [12,14]. It would seem once again therefore that the involvement of IL-6 is context dependent. Interestingly a potentially direct interaction between Rac1 and STAT3 has also been identified by co-immunoprecipitation with endogenous Rac1 from Rat1 lysates and exogenously expressed Rac1 in COS-1 cells, in addition to yeast two hybrid screens (Y2H) [22]. The interaction however was not observed in all cell types, again highlighting the cell type and context dependence of the Rac1–STAT3 relationship [14].

### MgcRacGAP

**Figure 4.**
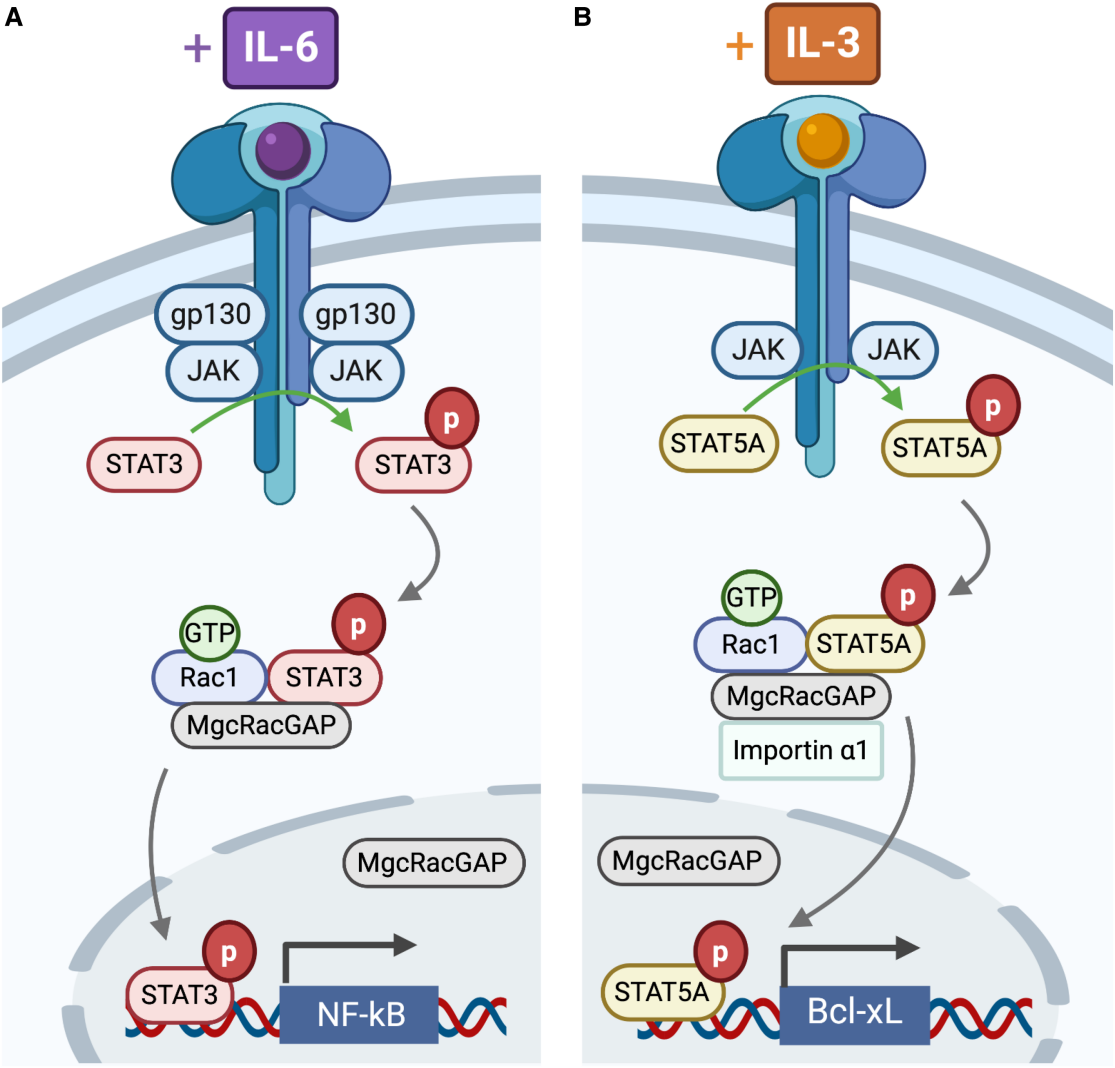
MgcRacGAP as a STAT nuclear chaperone. (**A**) IL-6 stimulation of murine M1 cells augments the formation of a STAT3, Rac1·GTP and MgcRacGAP ternary complex. Rac1 and MgcRacGAP are also required for nuclear import of pSTAT3, with MgcRacGAP translocating with pSTAT3. MgcRacGAP is also required for STAT3 driven transcription of NF-kB. (**B**) Similarly, IL-3 stimulation of Ba/F3 cells augments the formation of a STAT5A, Rac1·GTP and MgcRacGAP ternary complex. Both Rac1 and MgcRacGAP are also required for the nuclear translocation of pSTAT5A, with MgcRacGAP moving simultaneously with pSTAT5A. MgcRacGAP dependent nuclear import of STAT5 is shown to be reliant upon an interaction between the MgcRacGAP NLS and importin-α, a relationship not currently tested for STAT3. MgcRacGAP is required for STAT5A driven transcription of Bcl-xL.

A known regulator of Rac1–STAT signalling is the male germ cell Rac GTPase activating protein (MgcRacGAP) that has GAP activity towards Rac1 and Cdc42 [23]. MgcRacGAP was first identified by Y2H as a G12V Rac1 binding partner that repressed IL-6 stimulated differentiation of murine M1 and human HL-60 myeloid cells (Figure 4A) [24]. Deletion mutants indicated that the MgcRacGAP cysteine-rich domain (CRD) was essential for this repression. IL-6 stimulation of murine M1 cells was shown to augment co-immunoprecipitation of STAT3 with MgcRacGAP–Rac1 [25]. Additionally, it was shown that the GAP domain of MgcRacGAP is required for IL-6 induced STAT3 transcriptional activity in HeLa cells [25].

IL-3 stimulation of Ba/F3 (murine pro-B) cells similarly augmented co-immunoprecipitation of STAT5A with MgcRacGAP–Rac1 (Figure 4B), with the interaction involving the CRD and GAP domains of MgcRacGAP and the DBD of STAT5A [26]. The interacting region in the DBD was later further refined to residues 338–362 of STAT3 and the corresponding residues (341–365) of STAT5A, a conserved region in the STAT family [26]. A role for MgcRacGAP/Rac1 in the nuclear localisation of STAT3 and STAT5A has also been suggested after the concurrent nuclear translocation of pSTAT3 and MgcRacGAP in IL-6 treated M1 cells and pSTAT5A and MgcRacGAP in IL-3 treated Ba/F3 cells (Figure 4B) was demonstrated [25,26]. The dependence on Rac1 for such movement was confirmed by Rac1 knockout or expression of a dominant negative mutant [26]. Additionally, it was shown that STAT5 nuclear import in transfected MgcRacGAP conditional KO DT40 cells required an interaction between pTyrSTAT5A and importin-α, which was dependent on both active Rac1 and MgcRacGAP (Figure 4B). Although MgcRacGAP does not contain a classical nuclear localisation sequence (NLS), mutational analysis identified two basic sequences, 182KRR and 199KK, which can function as a bipartite NLS. Loss of this NLS abolishes pSTAT5A and pSTAT3 nuclear import, further demonstrating the importance of MgcRacGAP as a STAT nuclear chaperone [27].

Unexpectedly, MINC1, a small molecule inhibitor that stabilises the MgcRacGAP/Rac1 complex by inhibiting GAP-stimulated GTPase activity, exerts a contradictory, concentration-dependent activatory effect on STAT3 and STAT5A transcriptional activity in HeLa and HEK239 cells [28,29]. This could indicate that the GAP activity of MgcRacGAP is not essential for the induction of STAT transcriptional activity in this setting or that other MgcRacGAP domains are also contributing, for example the CRD. Indeed, the reliance on GAP activity of MgcRacGAP for STAT5A nuclear import in IL-3 induced Ba/F3 cells was not confirmed [26]. However, siRNA knockdown of MgcRacGAP led to increased STAT transcriptional activity, contradicting previous findings that MgcRacGAP stimulated IL-6-induced STAT3 transcriptional activity [25]. Additionally, MINC1 did not block STAT nuclear translocation in HEK293 SIE-Luc cells [28]. Overall these results have revealed that the relationship between MgcRacGAP, STAT activation and STAT nuclear movement is highly context and cell type dependent but could be crucial for mediating nuclear translocation of active STAT dimers to the nucleus.

### Biological function of the Rac1–STAT3 relationship

Many examples of Rac1-driven STAT3 activation are now known, with defects resulting in disease or dysfunction. Some pathways with known Rac1–STAT3 involvement are detailed in Table 1.

**Table 1:** Biological processes involving the Rac1/STAT3 signalling axis

Biological process	Description
**Mammary gland involution**	Death of epithelial cells after lactation. Rac1 and DOCK1 required for timely activation of STAT3, which triggers the start of involution and the activation of cell death pathways [30].
**Psoriasis**	Epidermal dysfunction disorder. Patients have elevated active Rac1 and consequently increased STAT3 activation [31].
**Chondrogenesis**	Development of cartilage. Rac1 driven STAT3 activation is required for this process [32].
**Mammalian primordial follicle pool production**	Pool production controls mammalian female reproductive abilities. Rac1 driven STAT3 activation is required for transcription of genes such as *Jagged1, BMP15, GDF9 and Nobox* [33].

As aberrant activation of STATs is observed in most cancer types, it is not surprising to find many examples of Rac1, and other Rho-family members, influencing STAT-dependent cancer progression. STAT3 has been shown to be activated by increased E-cadherin engagement in high density mouse epithelial HC11 cell populations, this was mediated by Rac1 activation [34,35] and led to cell survival. The Rac1–STAT3 relationship is also well documented to mediate invasion and proliferation and, in many cases, Rac1 acts upstream of STAT3, driving its activation. For example, Rac1 expression is elevated in breast cancer tumours and is linked to reduced patient survival. 3D cultures established from HCC1954 breast cancer cells treated with a small molecule Rac1 inhibitor had reduced pSer727 STAT3 levels and inhibited invasion [36]. Similarly, Rac1 is overexpressed in colorectal cancer (CRC), where Rac1 is shown to co-immunoprecipitate with STAT3 in HCT116 CRC cells [37]. As with breast cancer, chemical Rac1 inhibition impedes STAT3 activation and blocks epithelial-mesenchymal transition (EMT) in CRC [37]. Furthermore, 3D tumoursphere assays using glioblastoma cell lines expressing Rac1/2/3 showed increased numbers of tumourspheres (which relate to stem cell numbers) and increased STAT3 activation after IL-6 stimulation. Rac knockdown abolished STAT3 and ERK1/2 activation, and consequently impaired glioblastoma cell proliferation [38].

**Figure 5.**
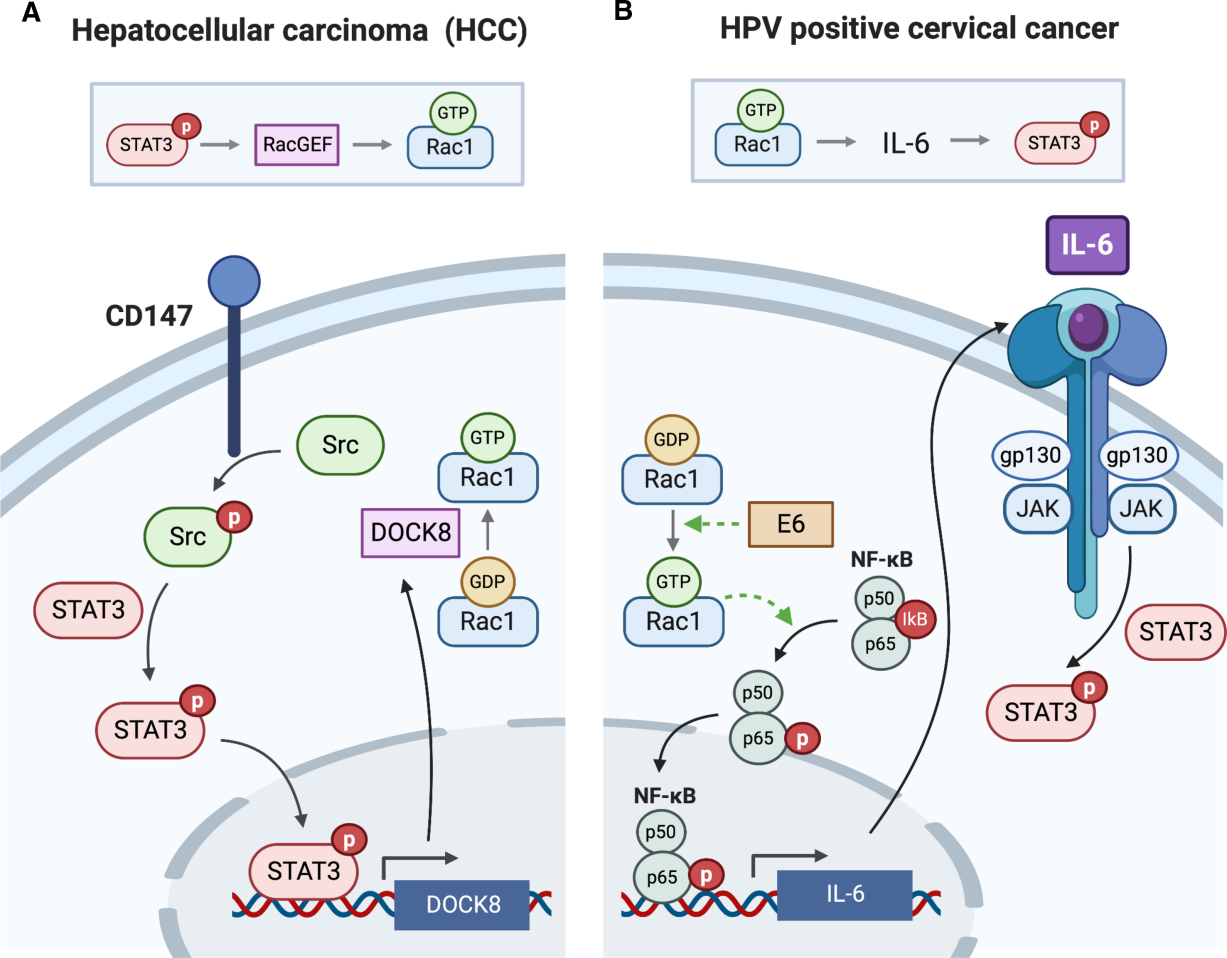
Rac1–STAT3/STAT3–Rac1 axes in cancer. (**A**) In hepatocellular carcinoma (HCC), STAT3 activation is the driver of Rac1 activation. Elevated HCC cell CD147 expression leads to increased Src phosphorylation and activation, which consequently triggers STAT3 phosphorylation and activation. STAT3 stimulates the transcription of *DOCK8*, a known RacGEF, thereby stimulating Rac1 activation and promoting metastasis. (**B**) In contrast, Rac1 is the driver of STAT3 activation in HPV positive cervical cancer cells. The E6 viral protein activates Rac1 through an unknown mechanism, which results in NF-κB activation involving p65 phosphorylation and AKT. NF-κB drives the increase in expression of IL-6, resulting in autocrine activation of STAT3, which is required for the proliferation and survival of cervical cancer cells.

However, in other cases, STAT3 has been shown to act upstream of Rac1, for example in the mesenchymal type movement of hepatocellular carcinoma (HCC) cells (Figure 5A). HCC cell expression of CD147, a glycosylated transmembrane protein, is required for the metastatic potential of HCC cells via activation of Src. Loss of CD147 or Src decreased Rac1·GTP levels, an effect transduced by STAT3. It was shown that Src-dependent STAT3 activation induced the transcription of a STAT3 target, *DOCK8*. DOCK8 is a Rac1 GEF, which stimulates Rac1 activation resulting in the cellular morphology changes required for HCC cell movement [39].

Human papillomavirus (HPV) positive cervical cancer cell lines have also been shown to have elevated pTyr705 STAT3 levels, which is driven by exacerbated IL-6 secretion (Figure 5B) [40]. It was shown that the oncogenic HPV protein, E6, is essential for mediating STAT3 activation in this setting. E6 expressing cells had elevated active Rac1 levels, which resulted in increased phosphorylation of the p65 subunit of NF-κB, a critical event required for nuclear translocation and transcription of the NF-κB target, IL-6 (Figure 5B). Rac1 activation of NF-κB has been previously described in the literature to involve the movement of IκB (the NF-κB inhibitor) to membrane ruffles where it is primed for degradation [41] however in this study only phosphorylation of p65 was monitored to assess activation, which was also shown to potentially involve AKT. The IL-6 expressed in HPV positive cervical cancer cells then facilitates autocrine STAT3 activation via JAK/IL-6R (Figure 5B). Rac1-driven activation of STAT3 therefore was shown to stimulate both the proliferation and survival of HPV positive cervical cancer cells, thus highlighting a further example of Rac1 driving upstream activation of STAT3 in cancer [40].

### Targeting the Rac1–STAT signalling node

A 240 amino acid small GTPase like protein fragment (SGLP) isolated from *Mycoplasma pulmonis* has been shown to induce cytoskeletal changes such as the formation of lamellipodia and filopodia, and increase Rac1 and STAT3 activation [42]. Critical to SGLP function is an N-terminal WxxxE motif, a sequence present in many bacterial virulence factors. Mutation of this motif abolished SGLP stimulated STAT3 activation by Rac1 and consequently blocked HeLa cell proliferation [42]. To investigate the therapeutic potential of the WxxxE motif, a 35mer peptide containing the WVLGE motif linked to an azurin cell penetrating peptide was synthesised [43]. This peptide could selectively target MCF7 cells over non-tumourigenic MCF-10A breast cancer cells. The peptide containing the motif alone however, acted as an inhibitor in comparison with the full SGLP virulence factor, with decreased Rac1 and STAT3 activation. The requirement for Rac1 was confirmed by co-expression with constitutively active Rac1, which overcame the peptide's inhibitory effect on STAT3 activation. Phenotypically, peptide-induced STAT3 inhibition decreased colony formation and reduced xenograft tumour growth [43], confirming that peptide inhibition of the Rac1–STAT3 axis could be of therapeutic utility.

ToxB is another bacterial protein that can influence Rac-driven STAT activation. *Clostridium difficile* ToxB is a known Rho-family inhibitor that, via Rac1 monoglucosylation and inhibition, induces cerebellar granule neurone (CGN) apoptosis [44,45]. This induction of apoptosis is known to be reliant on altered STAT family signalling [45]. Interestingly however it was shown that, although ToxB-induced Rac inhibition resulted in increased total STAT1 and pTyr705 STAT3 levels, neither were thought to contribute to CGN apoptosis [46]. Instead, ToxB treatment was shown to increase STAT5A activation and nuclear translocation, highlighting an unusual example of Rho family inhibition that results in STAT activation. By chromatin immunoprecipitation, STAT5A was shown to be enriched at the Bcl-xL promoter upon ToxB exposure, which resulted in transcriptional repression of this pro-survival signal but full mechanistic details are yet to be uncovered [46].

## Other Rho-family members

### RhoA

Constitutively active RhoA increases expression from STAT3- and STAT5A-driven luciferase reporters in HEK239T, BRL-4, CHO4, MDCK, MCF-7 and CHO cells, and stimulates both STAT tyrosine phosphorylation and DNA binding in transfected HEK239T and MDCK cells [13,15]. Expression of constitutively active RhoA in MEFs stimulates stress fibre formation and cell migration, which is lost upon STAT3 knockout [14]. RhoA expression is also elevated in CRC tumour samples, which results in increased pTyr705 STAT3 and increased CRC cell migration [47]. RhoA has also been implicated in haemopoietic cancers, such as B-cell precursor acute lymphoblastic leukaemia (BCP-ALL) where patients carry a TCF3-PBX1 fusion protein [48]. BCP-ALL cells have increased expression of Wnt16b that in turn stimulates total RhoA levels via a non-canonical Wnt signalling pathway. BCP-ALL cells additionally have increased expression of the tyrosine kinase-like orphan receptor, ROR1, which when silenced by shRNA results in reduced total RhoA and STAT3 levels, and impeded cell growth [48].

Additionally, STAT3 has been shown to inhibit RhoA in the process of astrogliosis, the formation of glial scars, with increased active RhoA observed in STAT3 knockout astrocytes [49]. It was shown that in wild type astrocytes, STAT3 can mediate the phosphorylation and activation of Ezrin, a RhoA antagonist, thus hindering RhoA activation in astrogliosis [49,50].

### RhoC

**Figure 6.**
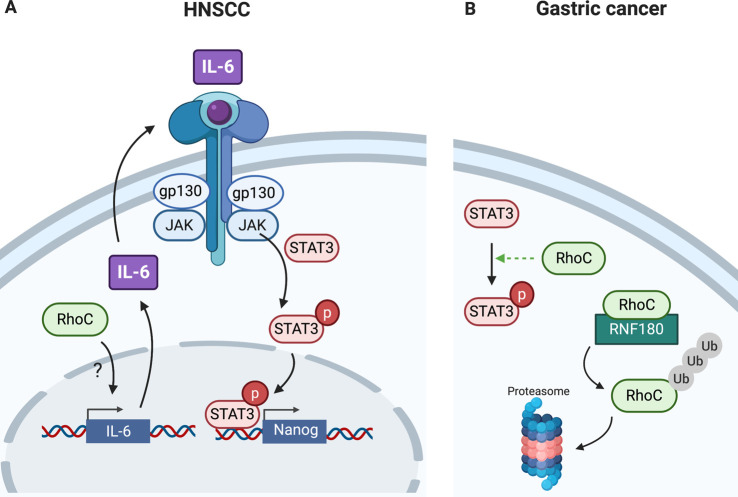
RhoC activation of STAT3 in cancer. (**A**) RhoC can stimulate IL-6 production in HNSCC cell lines, possibly via NF-κB activation, which allows for autocrine STAT3 activation via the IL-6R/JAK. Phosphorylated, activated STAT3 can then stimulate transcription of key transcription factors needed for cancer stem cell ‘stemness’. (**B**) RhoC is overexpressed in gastric cancer which increases STAT3 activation. Gastric cancer cells have low expression of the RhoC E3 ligase, RNF180, and thus increased expression of such ligase could help drive RhoC ubiquitination and proteasomal degradation, thus blocking STAT3 activation.

Constitutively active RhoC has been shown to activate STAT5A-driven transcription in HeLa and MCF7 cells [15]. Additionally, RhoC is known to influence STAT3 in cancer, a relationship that can be blocked by drugs that affect lipid modification and therefore membrane localisation and activation of Rho-family proteins. Inhibition of RhoC geranylgeranylation by atorvastatin caused inhibition of melanoma cell metastasis and reduced pSer727 STAT3 levels in a head and neck squamous cancer carcinoma (HNSCC) [51,52]. Additionally, RhoC promotes a cancer stem cell (CSC) phenotype in HNSCC [53]. shRNA knockdown of RhoC in HNSCC cell lines reduced the expression of core stem cell transcription factors including Nanog, Sox2 Oct3/4, all known transcriptional targets of STAT3. Overexpression of STAT3 in RhoC knockdown cells did not rescue the loss in stem cell transcription factor expression, confirming that RhoC is required as a STAT3 activator. Figure 6A details the interplay between STAT3 and RhoC in this setting. It has been shown that elevated RhoC expression in HNSCC drives increased IL-6 production by a currently unknown mechanism, postulated to involve NF-κB and the Rho effector, Rhotekin. The expressed IL-6 functions in autocrine activation of STAT3 via IL-6R/JAK. Activation of STAT3 results in the transcription of stem cell transcription factors required for the tumourigenic nature of CSCs [53].

Furthermore, RhoC activation of STAT3 has been recently implicated in gastric cancer, with elevated RhoC and pTyr705 STAT3 observed in tumour samples [54]. Gastric cancer cells have low expression of the RING finger E3 ligase, RNF180, which targets RhoC. It was shown that overexpression of RNF180 in gastric cancer cells can reduce STAT3 activation, attributed to increased RhoC proteasomal degradation (Figure 6B) [54]. This highlights how directed targeting of RNF180 to gastric cancer cells could be used to block RhoC-driven activation of STAT3.

### RhoH

RhoH is an atypical Rho-family protein [55] whose expression is limited to haematopoietic cells where it functions in T-cell development [56]. High RhoH expression is shown to decrease proliferation of IL-3 stimulated Ba/F3 cells where it differentially affects individual STAT family members [57]. RhoH overexpression results in increased STAT1 activation in IL-3 treated Ba/F3 cells, which induces the expression of p21*^cip1^* and p27*^kip1^*, two cyclin dependent kinase inhibitors, thus contributing to the impaired proliferative potential of these cells. Conversely, low RhoH expression results in increased STAT5A activation and increased transcription of a known STAT5A target, *IRF1*. Phenotypically, IRF1 expression results in the increased surface expression of CD123, which is a cell surface marker with elevated expression on acute myeloid leukaemia (AML) cells. A link between CD123 surface expression and RhoH expression was confirmed using the AML cell line, THP-1. As predicted, low RhoH expression in these cells was matched with elevated CD123 expression, which could be explained due to elevated STAT5A activation [57].

## Conclusions and comments

To date many examples of Rho-family small GTPase driven activation of STAT proteins have been identified that contribute to the proliferative and invasive capacity of many cancer cell types. Examples discussed in this review include Cdc42, Rac1, RhoA, RhoC and RhoH activation of oncogenic STAT3, and in some cases STAT5, in a range of cancers including but not limited to: breast, bladder, gastric, cervical, myeloid, pancreatic, hepatocellular, head and neck, and colorectal cancer. As well as the Rho-family serving as STAT activators, clear role reversal is also seen, where activated STAT proteins can lead to direct Rho-family activation. Many examples of Rho-family inhibition resulting in STAT de-activation have been discussed in this review. In some cases a direct relationship has been established, however compensatory mechanisms triggered by pathway cross-talk, leading to altered signalling cascades cannot be currently ruled out in some instances, confounding interpretation of some observations. Thus, a greater understanding is required of the complex Rho-STAT family relationship to aid successful therapeutic targeting of this important signalling network in cancer.

The data discussed here confirm non-canonical STAT activators as desirable therapeutic targets to ablate the constitutive STAT activation observed in the plethora of cancer types. Direct inhibition strategies for the Rho-family GTPases currently include targeting the GDP/GTP switching cycle or blocking the lipid modifications required for membrane localisation and activation [1]. However, many Rho-family inhibitors have failed to go beyond pre-clinical trials. As with other small G protein-driven pathways that have been identified as therapeutic targets, emphasis has shifted to the downstream effector proteins for Rho-family GTPases. Rho-family effectors that are kinases could be more suitable druggable targets [1,58]. Increasing numbers of Rho-family kinase effector-STAT relationships are being revealed, confirming the possibility of targeting this node in the pathway. For example, the RhoA effector ROCK2 is known to interact with STAT3 in human T cells and bind as a complex at STAT3 target promoters such as *IRF4* [59]. A Cdc42 specific effector, ACK, is also known to stimulate STAT1 and STAT3 activation in the presence of a cofactor, HSP90 [60]. PAK4, a dual Cdc42/Rac1 effector, is overexpressed in pancreatic CSCs and functions as an upstream activator of STAT3, which is required for pancreatic cancer stemness [61]. Additionally, PAK1 and PAK2 are known to interact with STAT5A in AML cell lines and stimulate STAT5A Ser779 phosphorylation [62]. Many Rho-family kinase effectors, such as the ROCK and PAK family, are serine/threonine kinases and thus greater understanding of the biological significance of STAT serine phosphorylation is required to confirm targeting these kinases as a suitable therapeutic strategy. The phosphorylation of the conserved STAT tyrosine residue has however been shown to be stimulated by ACK, the only tyrosine kinase effector of the Rho-family [63], further confirming the potential use of targeting this effector. To date, a handful of Rho-family kinase effector inhibitors have progressed to phase 1 clinical trials [1]. A 2014 trial using a first in class multi AGC kinase inhibitor, which targets ROCK1/2 was well tolerated in advanced solid tumours [64]. Additionally, a phase I trial targeting PAK4 in advanced solid tumours and non-Hodgkin lymphoma is currently ongoing (NCT02702492). Thus, targeting of Rho-family driven STAT activation could be achieved by tackling the effectors that relay Rho-family activation signals.

The STAT family are highly implicated in cancer formation and progression and successful targeting of their activators is sought. Continued research into Rho-family/STAT pathways should offer new and improved strategies for successful therapeutic intervention in STAT-driven cancers.

## Perspectives

- ***Importance of the field*:** STAT proteins are the master regulators of large transcriptional networks and their aberrant activation is observed in nearly all cancers; however, no STAT inhibitors exist in the clinic. Several JAK inhibitors, as a mode of indirect STAT inhibition, do have EMA and FDA approval but mainly for the treatment of rheumatoid arthritis, in addition to myeloproliferative neoplasms. However STAT inhibition in a wide spectrum of cancers still remains challenging [4].
- ***Current thinking*:** Rho-family GTPases, including Rac1 and Cdc42, have been shown to stimulate pathways that result in the activation of the oncogenic STATs, STAT3 and STAT5A. Rho-family driven activation of STATs has been shown to influence cancer cell proliferation, invasion and metastasis due to the stimulatory influence on pathways regulating cellular morphology, therefore, targeting these Rho-family-driven pathways should offer new therapeutic nodes of attack.
- ***Future directions*:** Further understanding of the signalling pathways involved in Rho-family driven STAT activation is required to successfully target such pathways. Direct Rho GTPase targeting is likely to be challenging, so more needs to be understood about the kinase effectors that likely link Rho GTPases to the STAT proteins, as these themselves could be tractable drug targets.

## Open Access

Open access for this article was enabled by the participation of University of Cambridge in an all-inclusive *Read & Publish* pilot with Portland Press and the Biochemical Society under a transformative agreement with JISC.

## Abbreviations

AML: acute myeloid leukaemia
AR-HIES: autosomal recessive hyper IgE syndrome
BCP-ALL: B cell precursor acute lymphoblastic leukaemia
CGN: cerebellar granule neurone
CRC: colorectal cancer
CRD: cysteine rich domain
CSC: cancer stem cell
DBD: DNA binding domain
EGFR: epidermal growth factor receptor
EMA: European Medicines Agency
EMT: epithelial-mesenchymal transition
ERK: extracellular signal regulated kinases
FDA: food and drug administration
GAP: GTPase activating protein
GDI: guanine nucleotide dissociation inhibitor
GEF: guanine nucleotide exchange factor
GPCR: G protein coupled receptor
HCC: hepatocellular carcinoma
HNSCC: head and neck squamous cancer carcinoma
HPV: human papillomavirus
HSP90: heat shock protein 90
IL: interleukin
JAK: Janus kinase
MAPK: mitogen-activated protein kinase
MEF: mouse embryonic fibroblast
Mgc: male germ cell
MM: multiple myeloma
NLS: nuclear localisation sequence
NTD: N-terminal domain
RING: really interesting new gene
SGLP: small GTPase like protein fragment
SH2: Src homology 2
shRNA: small hairpin RNA
STAT: signal transducer and activator of transcription
TAD: transactivation domain
Y2H: yeast two hybrid
